# From Approval to Evidence: Device Regulation, Long-Term Outcomes, and Conflicts of Interest in Metabolic Bariatric Surgery

**DOI:** 10.1007/s11695-026-08871-3

**Published:** 2026-07-20

**Authors:** Francesco Saverio Papadia

**Affiliations:** 1 University of Genoa, Genoa, Italy; 2 Ospedale Policlinico San Martino, Genoa, Italy

**Keywords:** Bariatric surgery, Medical devices, Gastric band, Gastric clip, Gastric electrical stimulation, Vagal blockade, Duodenal-jejunal bypass liner, Device regulation, Conflict of interest

## Abstract

**Background:**

Device-based innovation in metabolic bariatric surgery (MBS) has proliferated under evidentiary standards that differ substantially from those for pharmaceuticals. This review examines the lifecycle of MBS devices, from regulatory approval to long-term outcomes.

**Methods:**

We conducted a structured literature search of PubMed/MEDLINE, Embase, and Scopus for English-language publications from January 1990 through May 2026, supplemented by regulatory database searches (FDA, EUDAMED) updated to manuscript submission. Five device categories representing distinct regulatory pathways were selected a priori: adjustable gastric bands (LAGB), implantable gastric stimulation (IGS) and vagal blockade (VBLOC), endoluminal liners (EndoBarrier), and gastric clips (BariClip). Intragastric balloons were excluded as temporary, non-implanted devices with different regulatory and safety profiles. This is a narrative review that used systematic search methods for transparency; it does not claim to be a systematic review or meta-analysis.

**Results:**

The LAGB received FDA approval in 2001 based on 3-year data showing 36.2% excess weight loss (EWL), despite an advisory panel voting 6 − 4 that 2-year data were inadequate. Ten-year follow-up from the Teen-LABS study (2025) demonstrated that, among adolescents with retained bands, median BMI was 51.1, a 9.2% increase from baseline in this adolescent cohort. Implantable gastric stimulation failed to show benefit in the sham-controlled SHAPE trial (11.8% vs. 11.7% EWL, *p* = 0.717). Vagal blockade (VBLOC) showed equivocal results in the ReCharge trial (26.1% vs. 16.9% EWL, missing its prespecified margin) yet received FDA approval in 2015 via the Humanitarian Device Exemption (HDE #H150003). The EndoBarrier received CE marking in 2009 and was widely adopted before being withdrawn; a subsequent rigorous sham-controlled trial (ENDO, 2025) confirmed efficacy but revealed a 3.3% hepatic abscess rate. The BariClip, lacking FDA approval and an active MDR CE mark (EUDAMED search performed April 15, 2026, found no active certificate), has been studied only in small single-center series with short-term follow-up (6–24 months).

**Conclusions:**

The history of device innovation in MBS reveals a recurring pattern: regulatory approval or market entry based on limited evidence, followed by gradual recognition of limited efficacy and device-specific complications. As noted in a companion systematic review, these failures are not limited to devices but reflect a broader systemic challenge. Structured oversight, including prospective registration, sham-controlled trials where feasible, post-market registries, and long-term follow-up requirements, is essential to protect patients and preserve the credibility of the field.

## Key Points

A recurring pattern across MBS devices (LAGB, IGS, VBLOC, EndoBarrier, BariClip) shows regulatory approval or market entry based on limited evidence, followed by late recognition of limited efficacy and device-specific complications, from the LAGB’s 6–4 approval vote (October 2000) and weight regain in adolescents (9.2% BMI increase at 10 years, though this finding is specific to the adolescent cohort) to the EndoBarrier’s 3.3% hepatic abscess rate identified only after a post-withdrawal sham-controlled trial.

Procedure-specific devices create concentrated conflicts of interest (manufacturer’s survival tied to a single operation), unlike general surgical instruments. This requires structural management beyond disclosure alone.

Recommended reforms for procedure-specific devices include independent data monitoring committees, separation of conflicted investigators from safety analyses, sham-controlled trials where feasible, and mandatory long-term post-market registries.

## Introduction

Surgical innovation has always been intertwined with device development. In metabolic bariatric surgery (MBS), this relationship is particularly intimate: adjustable gastric bands, electrical stimulators, vagal blockers, intraluminal liners, and gastric clips have each promised weight loss with less anatomical disruption than traditional operations [1–3]. 

Yet device innovation occupies a contested regulatory space. Unlike pharmaceuticals, which require large randomized trials before approval, many devices enter clinical use through pathways requiring only demonstration of “substantial equivalence” to an existing device [4]. The 510(k) pathway, designed for iterative improvements to low-risk devices, has been used to introduce novel technologies with unproven clinical benefit [5]. In the European Union, the transition from the Medical Device Directive (MDD) to the new Medical Device Regulation (MDR) has left many legacy devices in regulatory limbo [6]. 

This asymmetry has real consequences. A new obesity drug must demonstrate safety and efficacy in large, well-controlled trials [7]. A new surgical device may enter clinical use based on mechanical similarity to a predicate device whose clinical performance was marginal. Device failures, like erosion, migration, infection, obstruction, often require multiple revisional surgeries, each with additive risk [8]. 

This narrative review examines the lifecycle of device-based innovations in MBS through five representative case studies. Drawing on the IDEAL framework for surgical innovation [9] and the IFSO position statement on innovative procedures [10], we analyze how device oversight has succeeded and failed and propose a framework for improvement. The failures documented here for device-based innovations are not isolated: a companion systematic review of non-standard intestinal-based MBS procedures (co-authored by members of the current author team) found that 89% were introduced without prospective trial registration and that over 10,000 patients underwent innovative operations outside a transparent research framework [38]. 

## Methods: Search Strategy and Case Selection

We conducted a structured literature search of PubMed/MEDLINE, Embase, and Scopus for English-language publications from January 1990 through May 2026. Search terms included: “adjustable gastric band,” “laparoscopic gastric banding,” “gastric electrical stimulation,” “implantable gastric stimulator,” “vagal nerve blockade,” “VBLOC,” “duodenal-jejunal bypass liner,” “EndoBarrier,” “gastric clip,” and “BariClip.” FDA summaries of safety and effectiveness data and the publicly accessible EUDAMED database were reviewed; regulatory database searches were updated to the date of manuscript submission (April 15, 2026), independently of the literature search end date.

Five device categories were selected a priori to represent distinct regulatory pathways (PMA, HDE, CE mark under MDD, no active MDR mark) and distinct mechanisms of action (restrictive, electrical, vagal, endoluminal). Intragastric balloons were excluded from this review because they are temporary, non-implanted devices that follow different regulatory pathways (primarily FDA 510(k) or De Novo) and have a distinct safety profile centered on balloon deflation, pancreatitis, and gastric perforation. Their inclusion would have diluted the focus on permanently or long-term implanted, procedure-specific devices.

This is a narrative review that used systematic search methods for transparency in case identification. We do not claim to have performed a systematic review or meta-analysis. The search was designed to identify landmark trials, regulatory decisions, and long-term follow-up studies for each prespecified device category. Where randomized controlled trial data were available, these were prioritized over observational series. No formal screening log or PRISMA flow diagram was kept, consistent with the narrative design.

Implantable gastric stimulation and vagal blockade are treated separately in the results given their different outcomes, though they share mechanistic proximity.

### The Regulatory Landscape for Surgical Devices

In the US, medical devices fall into three risk classes. Class III devices require Premarket Approval (PMA) with clinical evidence of safety and effectiveness. However, many MBS devices have entered via the **510(k) pathway**, requiring only demonstration of “substantial equivalence” to an existing predicate device. This standard has been criticized for allowing iterative changes to accumulate into substantial clinical differences without new clinical evaluation [5]. The **De Novo pathway** is available for novel low-to-moderate risk devices. The **Humanitarian Device Exemption (HDE)** allows approval based on “probable benefit” for conditions affecting fewer than 8,000 US patients annually (the threshold was updated to 8,000 in 2016; previously 4,000). This pathway has been controversially used for obesity devices.

In the EU, the **Medical Device Regulation (MDR) 2017/745** has tightened requirements substantially, but for years the CE marking pathway under the predecessor MDD was less demanding than even the 510(k) process, contributing to device proliferation without robust outcomes data [6]. 

The **IDEAL framework** (Idea, Development, Exploration, Assessment, Long-term study) provides a structured pathway for surgical innovation [9, 11]. Originally published in 2009 and updated in 2019, its application to MBS devices has been inconsistent, as illustrated by the case studies below.

## Case Study 1: The Adjustable Gastric Band — A Cautionary Benchmark

The LAP-BAND received FDA approval on June 5, 2001, under PMA number P000008 [12]. The pivotal trial enrolled 299 patients with 36-month follow-up. Mean %EWL was 36.2%, and 15% required explantation within 3 years. Crucially, the FDA advisory panel voted 6–4 against recommending approval in 2000, citing inadequate 2-year follow-up data. The FDA approved the device after receiving 3-year data, *without reconvening the panel* (FDA meeting minutes, archived at docket FDA-2000-N-0023) [12]. 

Early post-approval studies raised concerns. DeMaria and colleagues reported 41% device removal and 71% esophageal dilatation at 36 months [13]. A later Medicare study of 25,042 beneficiaries found 18.5% required reoperation, averaging 3.8 procedures per patient [8]. 

The Teen-LABS 10-year follow-up (2025) provided important long-term data specific to adolescents [14]. Of 14 adolescents who received LAGB, only 8 retained their bands at 10 years. Among those 8 patients, median BMI was 51.1, representing a 9.2% increase from baseline. There was no durable improvement in comorbidities. While these findings are compelling, they apply specifically to the adolescent population; adult registry data show high reoperation rates but not the same degree of weight regain to above-baseline levels.

The LAGB represented a novel phenomenon in bariatric surgery: a device designed, manufactured, and marketed exclusively for a single procedure on a mass scale. Unlike multi-use instruments, like staplers, laparoscopic equipment, energy devices, which have many indications across many surgical specialties, the LAGB had no purpose other than gastric banding. Its commercial success depended entirely on the continued adoption of that single operation. This created a structural financial incentive for the manufacturer and for surgeon-investigators with equity or royalty ties. The advisory panel’s 6–4 vote against approval, overridden by FDA staff, reflected tensions that were as much commercial as scientific. The LAGB thus exemplified the concentrated conflict of interest inherent to procedure-specific devices: a single-use device, a massive potential market, and regulatory pathways that did not require long-term outcomes data before widespread adoption.

A balanced historical assessment of the LAGB is warranted. The band played a pivotal role in popularizing laparoscopic bariatric surgery in the early 2000 s, offering a technically simpler, reversible, and less anatomically disruptive alternative to gastric bypass. It expanded access to surgical weight loss and trained a generation of minimally invasive bariatric surgeons. However, as longer-term data accumulated, it became clear that the band’s efficacy was not durable for many patients, and device-specific complications (slippage, erosion, esophageal dilatation) were more common than initially appreciated. In this sense, the LAGB may be best understood as the “wrong operation at the right time”, i.e. a procedure that advanced the field conceptually and technically but whose limitations became apparent only after widespread adoption. Acknowledging this historical contribution does not diminish the obligation to learn from the band’s failures; rather, it highlights the need for better evidentiary standards before widespread adoption. Its market share has since collapsed from approximately 35% of bariatric operations in the United States in 2008 to less than 2% by 2018 [15, 16]. 

## Case Study 2: Implantable Gastric Stimulation — When Sham Controls Work

The concept of gastric electrical stimulation for obesity originated in the 1990s. Open-label studies showed promise [17, 18]. However, the pivotal SHAPE trial (2009), a prospective, randomized, double-blind, sham-controlled study of 190 patients, found no significant difference between active and sham groups (11.8% vs. 11.7% EWL, *p* = 0.717) [19]. The device was never FDA-approved for obesity, representing a success for rigorous evaluation: a sham-controlled trial prevented widespread adoption of an ineffective therapy before it could cause population-level harm.

## Case Study 3: Vagal Nerve Blockade (VBLOC) — Equivocal Evidence, Controversial Approval

The VBLOC system had a more ambiguous trajectory. The EMPOWER trial (Sarr et al., 2012; 294 patients) showed a non-significant numerical difference in %EWL between active and sham groups at 12 months; the trial was stopped early following an interim analysis [20]. The ReCharge trial (Ikramuddin et al., 2014) showed a larger difference (26.1% vs. 16.9% EWL) that nonetheless did not meet the prespecified 10-point non-inferiority margin [21]. A further 18-month follow-up confirmed sustained separation between groups [22]. 

Despite these equivocal results, the device received FDA approval on January 12, 2015, via the **Humanitarian Device Exemption pathway (HDE #H150003)**. The HDE is intended for conditions affecting fewer than 8,000 US patients annually. The controversy is not about the numerical threshold itself (currently 8,000) but about how the target population was defined in the VBLOC application. The sponsor requested approval for a narrow subpopulation of patients with obesity who had failed non-surgical weight loss attempts, thereby artificially constraining the denominator to meet the 8,000-patient cap. This application of the HDE pathway to a condition that affects tens of millions of Americans has not been critically examined in the literature. The HDE requires only “probable benefit” rather than proven effectiveness, a substantially lower evidentiary bar than PMA. Whether this approval accurately reflected the intended scope of the exemption merits independent regulatory review.

## Case Study 4: The Duodenal-Jejunal Bypass Liner — Efficacy, then Withdrawal

The EndoBarrier (GI Dynamics), an endoscopic intestinal liner, received CE marking in 2009 and was widely adopted in Europe and Australia based on limited early data [23–25]. In 2017, GI Dynamics withdrew the device from the market, citing commercial and financial constraints rather than a safety signal. At that point, no rigorous sham-controlled trial had been completed.

The ENDO trial (Thompson et al., 2025) was a multicenter, double-blind, randomized, sham-controlled trial of 320 patients with type 2 diabetes and obesity that was initiated *after* the 2017 market withdrawal [26]. This unusual sequence, widespread clinical adoption preceding the pivotal trial, which was then completed after commercial withdrawal, strikingly illustrates the risks of under-evidenced regulatory approval.

The DJBL group achieved significant HbA1c reduction (1.10% vs. 0.28%, *p* = 0.0004) and weight loss (7.7% vs. 2.1% TWL). However, the study was halted early due to a 3.3% hepatic abscess rate (7/212 patients). A concurrent registry study of 929 patients reported a lower rate (1.1%), suggesting that procedural modifications may reduce but not eliminate this risk [27]. 

### Case Study 5: The BariClip — An Emerging Pattern

The BariClip (Advanced Bariatric Technology, LLC) is a non-adjustable, vertical gastric clip made of titanium and silicone [28, 29]. It has not received FDA approval. Regarding European regulatory status: a search of the publicly accessible EUDAMED database was last performed on the date of manuscript submission (April 15, 2026), and no active MDR certificate was found for this device. Legacy MDD certification cannot be excluded, but any such certification would predate the current, more stringent regulatory framework.

Published evidence consists of small, single-center, retrospective case series with follow-up of 6–24 months. Reported outcomes include %EWL of 65–74% at 6–24 months, slippage rates of 2.4–4.5%, and erosion rates of 1–2% [30–33]. Notably, Bonaldi and colleagues concluded that BariClip gastroplasty remains an experimental procedure requiring cautious evaluation [33]. 

Revision after gastric clipping is technically demanding. Chang and colleagues reported six patients who underwent revision, with a mean operative time of 286 min; laparoscopic instruments broke during clip removal in three cases [34]. The claim of reversibility is therefore meaningful only with appropriate surgical infrastructure and patient counseling.

The long-term behavior of any rigid, non-degradable foreign body placed in sustained contact with the gastric wall warrants vigilance. The history of foregut devices, including the Angelchik anti-reflux prosthesis, in which erosion was documented up to 40 years post-implantation [35, 36], underscores that current follow-up of 6–24 months is insufficient to characterize late complication rates. The BariClip differs meaningfully in design and placement from prior foregut implants, but the general principle applies: late device-tissue interactions cannot be excluded on the basis of short-term series alone.

## Cross-Cutting Themes and Discussion

### The Recurring Pattern

Across device categories, a consistent structural pattern emerges: early promise from small, single-arm series; regulatory approval or market entry based on limited evidence; and gradual recognition of limited efficacy or device-specific complications as follow-up lengthens. The LAGB, EndoBarrier, and BariClip (to date) follow this trajectory. The IGS is the important exception, as it was eliminated by a sham-controlled trial before widespread adoption, demonstrating that rigorous early evaluation can prevent population-level harm.

This pattern is not unique to devices. A companion systematic review of 57 non-standard intestinal MBS procedures (first-in-human or early series, excluding devices) found that the median time from first operation to publication was 5 years, only 11% of studies had prospective trial registration, and 98% were judged at serious overall risk of bias [38]. Across over 10,000 patients who underwent these innovative procedures, the same structural failures were documented: delayed reporting, absent registration, inadequate follow-up. The device-specific cases presented here thus reflect a broader systemic challenge in surgical innovation governance.

### Why does this Pattern Persist?

Several factors contribute. First, regulatory asymmetry: devices face a lower evidentiary bar than drugs. Second, financial incentives: manufacturers and surgeon-inventors may hold equity or consulting relationships that create pressure for speed-to-market. Third, patient vulnerability: patients seeking less invasive options may accept higher long-term risks based on incomplete information. Fourth, publication bias: positive early results are published; null and negative results may remain unpublished. Fifth, inadequate long-term follow-up: late complications go unrecognized until a device achieves sufficient adoption to generate population-level data, by which time harm may already be widespread.

### Managing Conflicts of Interest: Procedure-Specific Devices vs. General Instruments

The IFSO position statement explicitly identifies conflict of interest, both financial and academic, as a key concern in device innovation [10]. However, not all device-related conflicts are equivalent. A critical distinction has been overlooked in the surgical literature: the difference between *multi-use general surgical instruments* and *procedure-specific devices*.

Staplers, laparoscopic towers, energy devices, and robotic systems are used across hundreds of procedure types and multiple specialties. A surgeon’s preference for one brand over another, while subject to industry influence, is diffused across many clinical contexts. The financial incentive is diluted: no single procedure’s success or failure determines the device’s commercial fate.

The LAGB, by contrast, represented a device designed, manufactured, and marketed *exclusively for a single procedure on a mass scale*. Its commercial success depended entirely on the continued adoption of gastric banding. This created not a presumption of misconduct, but an inherent vulnerability: the manufacturer’s financial survival was tied to the band’s market penetration, and surgeon-investigators who developed or popularized the device often had equity, consulting, or royalty relationships. We do not assert that any specific manufacturer or surgeon acted improperly; rather, we argue that the structure itself creates pressures that can distort evidence generation and interpretation unless managed with independent oversight.

This concentrated structural incentive applies to other procedure-specific devices examined in this review. The BariClip is a single-use clip with no other indication; its commercial fate depends entirely on the adoption of gastric clipping. The EndoBarrier had no use other than intestinal liner placement. The VBLOC system had no use other than vagal blockade for obesity. Each of these devices shares the same structural vulnerability: a manufacturer whose survival depends on a single procedure, and surgeon-investors whose financial interests align with that procedure’s success.

**Adequate management of procedure-specific device conflicts** requires more stringent safeguards than those applied to general surgical instruments. We propose:

1. **Independent Data Monitoring Committees (IDMCs)** with no financial ties to the manufacturer, empowered to halt trials for safety or futility. This was successfully implemented in the ENDO trial [26].
2. **Separation of data analysis** from investigators with equity holdings. Independent statisticians should perform primary analyses.
3. **Mandatory prospective disclosure** of all investigator financial relationships (equity, consulting fees, patents, royalties) at trial registration and publication.
4. **Public registries of surgeon-industry ties** specific to procedure-specific devices, maintained by professional societies such as IFSO.
5. **Investor recusal from safety adjudication**: Surgeons with equity in a procedure-specific device should not serve as the sole arbiters of safety data. IDMCs should have this authority.
6. **Long-term post-market registries** with mandatory participation for all patients receiving procedure-specific devices, as a condition of device approval.

These requirements are consistent with CONSORT guidance for non-pharmacological trials [37] and extend the IFSO position statement’s recommendations [10]. The question is not whether conflicts exist, as these are inherent to surgical innovation, but whether they are managed through structural mechanisms rather than disclosure alone. For procedure-specific devices, disclosure is insufficient; independent oversight is essential.

### Realistic Implementation: what can be Done Now vs. what Requires Regulation

The six reforms differ in enforceability. Some can be implemented immediately by journals and professional societies; others require regulatory or payer action.

### Immediate Actions (Journals, Societies, IRBs)

- Journals can require prospective disclosure of equity, consulting, and royalties as a condition of publication.
- Journal editors can demand that trial protocols specify who performed safety analyses and whether any investigator with equity had access to unblinded data.
- IFSO/ASMBS could launch public, searchable registries of surgeon-device financial ties within 12 months.
- IRBs can require investor recusal from safety adjudication (i.e., independent committee, not conflicted investigators).

### Longer-Term Actions (Regulators, Payers, 2–5 Years)

- FDA and EU notified bodies could mandate IDMCs for all PMA, HDE, and Class III device studies.
- CMS and private payers could condition reimbursement on participation in mandatory post-market registries (modelled on the Michigan Bariatric Surgery Collaborative).

These proposals are feasible: components already work in orthopedics and cardiac surgery. The bariatric community can adopt the immediate actions today without waiting for new legislation.

### The Value of Sham-Controlled Trials

The SHAPE, EMPOWER, ReCharge, and ENDO trials demonstrate that sham-controlled trials are feasible and essential in device research. The SHAPE trial prevented adoption of an ineffective therapy. The ENDO trial identified a hepatic abscess rate that retrospective series had under-detected. These trials are not obstacles to innovation; they are how innovation becomes knowledge.

### Toward a Better Framework

The IDEAL framework provides a validated roadmap for staged surgical innovation [9, 11]. Applying it to MBS devices requires adherence to several core principles: prospective registration of all device studies, independent data monitoring from Stage 2a onward, sham-controlled trials where ethically feasible, minimum follow-up durations that reflect the latency of device-specific complications (the Angelchik experience suggests that 5 years may still be insufficient), and mandatory post-market registries with long-term surveillance. The critical gap between existing guidance and current practice is not the absence of frameworks but the failure to enforce them.

Additional requirements applicable to MBS devices should include: mandatory reporting of all device-related complications and reoperations using standardized classification systems; periodic regulatory re-evaluation with authority to withdraw approval based on post-market data; and informed consent explicitly addressing regulatory status, evidence limitations, and the technical demands of revision surgery (Table 1).

**Table 1:** Comparison of five procedure-specific MBS devices

Device	Regulatory pathway (year)	Pivotal trial evidence	Long-term outcomes (≥ 5 years)	Current status	Key device-specific complications
LAGB (LAP-BAND)	FDA PMA (2001)	3-year: 36.2% EWL, 15% explant [12]	Teen-LABS (adolescents): 9.2% BMI increase at 10 years [14]; adult reoperation 18.5% [8]	< 2% of procedures [15, 16]	Slippage, erosion, esophageal dilatation, port/tube issues
IGS (gastric stimulator)	None – failed SHAPE	Sham-controlled: 11.8% vs. 11.7% EWL, *p* = 0.717 [19]	No long-term data (never approved)	Not FDA-approved, not commercially available	Not applicable
VBLOC (Maestro)	FDA HDE #H150003 (2015)	ReCharge: 26.1% vs. 16.9% EWL (did not meet 10-point margin) [21]	18-month sustained separation [22]; no 5-year data	Commercial failure, not widely adopted	Pain at neuroregulator site, lead migration
EndoBarrier	CE mark (2009); withdrawn 2017	ENDO trial (2025): 7.7% vs. 2.1% TWL, HbA1c reduction 1.10% vs. 0.28% [26]	None beyond 3 years; registry showed 1.1% abscess [27]	Not commercially available	Hepatic abscess (3.3% in ENDO), device migration, GI bleeding
BariClip	No FDA approval; no active MDR CE mark (EUDAMED search 4/15/2026)	Single-center series, 6–24 mo: 65–74% EWL [30–33]	None beyond 24 months	Experimental per own investigators [33]	Slippage (2.4–4.5%), erosion (1–2%), difficult revision [34]

### Limitations

This is a narrative review that used structured search methods for transparency; it is not a systematic review or meta-analysis. No PRISMA flow diagram or formal risk of bias assessment was performed. Case selection, while representative, is not exhaustive. Recent withdrawals of intragastric balloon devices (Obalon, ReShape Duo) following FDA safety communications, and the commercial failure of the VBLOC Maestro system, suggest that the pattern extends across the broader MBS device landscape. The Teen-LABS LAGB conclusions derive from a small retained-band cohort (*n* = 8) and apply specifically to adolescents; adult data show high reoperation rates but not the same degree of weight regain. The ENDO trial is currently Epub ahead of print [26]. Finally, while this review focuses on devices, the oversight failures described here are corroborated by a companion systematic review of procedural innovations in MBS [38].

## Conclusions

The history of device innovation in MBS reveals a recurring pattern: regulatory approval or market entry based on limited evidence, followed by late recognition of limited efficacy and device-specific complications. The LAGB, a single-use device with a massive market, popularized laparoscopic bariatric surgery but ultimately showed limited durability. The IGS was correctly prevented from adoption by a sham-controlled trial. VBLOC received controversial HDE approval despite equivocal evidence. The EndoBarrier was widely adopted under CE marking, withdrawn for commercial reasons, then found in a post-hoc rigorous trial to have a 3.3% hepatic abscess rate. The BariClip, with ambiguous regulatory status and short-term retrospective evidence, is following the early stages of this pattern; its own investigators describe it as experimental.

These are systemic failures of regulatory pathways, professional oversight, and research culture. As noted in a companion systematic review [38], these failures are not limited to devices but reflect a broader systemic challenge. The solution is not to abandon innovation but to subject it to rigorous standards: prospective registration, sham-controlled trials where feasible, long-term follow-up, and post-market registries enforced through regulatory and accreditation requirements. Addressing these failures requires structural reforms: independent data monitoring, separation of conflicted investigators from primary safety analyses, and mandatory prospective registration with public disclosure of all financial relationships. For procedure-specific devices, disclosure alone is insufficient. Professional societies, regulators, journal editors, and surgeons must recognize that patient safety depends on evidence, not enthusiasm.

## Data Availability

No datasets were generated or analysed during the current study.
